# Genetic Variant in *MTRR*, but Not *MTR*, Is Associated with Risk of Congenital Heart Disease: An Integrated Meta-Analysis

**DOI:** 10.1371/journal.pone.0089609

**Published:** 2014-03-04

**Authors:** Bingxi Cai, Ti Zhang, Rong Zhong, Li Zou, Beibei Zhu, Wei Chen, Na Shen, Juntao Ke, Jiao Lou, Zhenling Wang, Yu Sun, Lifeng Liu, Ranran Song

**Affiliations:** 1 Department of Epidemiology and Biostatistics and State Key Laboratory of Environment Health (Incubation), MOE (Ministry of Education) Key Laboratory of Environment and Health, Ministry of Environmental Protection Key Laboratory of Environment and Health, School of Public Health, Tongji Medical College, Huazhong University of Science and Technology, Wuhan, China; 2 Department of Maternal and Child Health, School of Public Health, Tongji Medical College, Huazhong University of Science and Technology, Wuhan, China; South Texas Veterans Health Care System and University Health Science Center San Antonio, United States of America

## Abstract

**Background:**

Congenital heart disease (CHD) is one of the most common birth defects and the leading cause of deaths among individuals with congenital structural abnormalities worldwide. Both Methionine synthase reductase (*MTRR*) and Methionine synthase (*MTR*) are key enzymes involved in the metabolic pathway of homocysteine, which are significant in the earlier period embryogenesis, particularly in the cardiac development. Evidence is mounting for the association between *MTRR* A66G (rs1801394)/*MTR* A2756G (rs1805087) and the CHD risk, but results are controversial. Therefore, we conducted a meta-analysis integrating case-control and transmitted disequilibrium test (TDT) studies to obtain more precise estimate of the associations of these two variants with the CHD risk.

**Methods:**

To combine case-control and TDT studies, we used the Catmap package of R software to calculate odds ratios (ORs) and 95% confidence intervals (CIs).

**Results:**

A total of 9 reports were included in the final meta-analysis. Eight of them comprised of 914 cases, 964 controls, and 441 families that were germane to *MTRR* A66G polymorphism; and 4 reports comprised of 250 cases, 205 controls, and 53 families that were relevant to *MTR* A2756G polymorphism. The pooled OR for the *MTRR* 66 G allele versus A allele was 1.35 (95% CI = 1.14–1.59, *P*<0.001, *P*_heterogeneity_ = 0.073). For *MTR* A2756G, the G allele conferred a pooled OR of 1.10 (95% CI = 0.78–1.57, P = 0.597, *P*_heterogeneity_ = 0.173) compared with the A allele. Sensitivity analyses were carried out to asses the effects of each individual study on the pooled OR, indicating the stability of the outcome. Moreover, positive results were also obtained in all subgroups stratified by study type and ethnicity except the subgroup of TDT studies in *MTRR* A66G variant.

**Conclusions:**

This meta-analysis demonstrated a suggestive result that the A66G variant in *MTRR*, but not the A2756G in *MTR*, may be associated with the increase of CHD risks.

## Introduction

Congenital heart disease (CHD), an abnormality in the heart's structure or function that arises before birth [1], is characterized by defects in the cardiac architecture that interfere with venous drainage, septation of cardiac segments and their sequences, and regular function of valve apparatuses [2]. CHD can be classified into three broad categories: cyanotic heart disease, left-sided obstruction defects and septation defects [1] It represents one of the most common birth defects and the leading cause of death from a congenital structural abnormality worldwide affecting approximately 8 per 1000 live births reported by America Heart Association [3]. In China, the prevalence increased sharply from 1.2 to 5.4 per 1,000 live births between 2000 and 2011, making it the number one among all birth defects [4]. Patients with CHD require treatments may be a clinical and economic burden as nearly a quarter of them will need surgery in the first year of life and they are also considered part of a neurological and psychomotor abnormalities and malnutrition high-risk group [5], [6], [7]. CHD has become a serious public-health issue and more efforts are needed to lucubrate.

Current knowledge on susceptibility, as well as pathological mechanism underlying the development of CHD has indicated a multiple exposure interaction between genetic and environmental factors [8]. Folic acid, a form of water-soluble vitamin B_9_, plays an important role during embryologic development, has been shown to prevent CHD during pregnancy [9]. Catalyzed by various enzymes, especially *MTRR* and *MTR*, folic acid aid in converting homocysteine into methionine. It is widely accepted that the occurrence of CHD relies not only on the insufficiency of folic acid [10], but also on the hyperhomocysteinaemia (Hcy) causing by genetic defect in folate metabolism pathway. Evidence shows that Hcy is associated with a high risk of heart defect incidence [11], which implies an appreciable genetic component of single nucleotide polymorphisms (SNPs) in folate pathways in these disorders [12]. Furthermore, SNPs with the function that might inhibit the folate pathway were reported to be involved in pathogenesis of neural tube defect and cancers [13], [14].

Methionine synthase (*MTR*) and methionine synthase reductase (*MTRR*), major regulatory enzymes involved in homocysteine metabolic pathway, map to chromosomes 1q43 and 5p15.31 respectively. *MTR* catalyzes the remethylation of homocysteine to form methionine, which is the decisive step in methionine biosynthesis [15]. *MTRR* is responsible for activating methionine synthase via reductive methylation modulating a functional deficiency of methionine synthase when *MTRR* activity is disrupted [16], [17]. In the midst of all polymorphisms of these two genes, *MTRR* A66G (rs1801394) and *MTR* A2756G (rs1805087) were discussed mostly as a genetic cause of CHD in humans. *MTRR* A66G polymorphism results in an isoleucine-to-methionine substitution, whose biochemical phenotypes alter the affinities for the redox partner—methionine synthase. Likewise, *MTR* A2765G is a missense mutation causing a codon of aspartate substituted by a glycine residue. [18].

The association between *MTRR* A66G/*MTR* A2756G and the risk of CHD has been reported among numerous studies [19], [20], [21], [22], [23], [24], [25], [26], [27]. However, despite these hard won successes begin to shed light on pathogenic mechanisms of CHD, such approaches have struggled to provide replicable results for the association. The transmission/disequilibrium test (TDT) based on family is particularly advantageous as less confounding caused by population admixture and it is of the same importance as case-control study in genetic association analysis. Integrating case-control and TDT studies will enhance the statistical power with the enlargement of sample size, which is limited in individual observations, and provide an overall picture of the effect size attributable to genetic polymorphism. Therefore, we conducted a meta-analysis to integrate the results from case-control and TDT studies to estimate the association of *MTRR* A66G and *MTR* A2756G with CHD risk more accurately.

## Materials and Methods

### Search strategy

We searched the relevant literatures from Medline, ISI Web of Science, China National Knowledge Infrastructure (CNKI) and Sinomed databases before March 1, 2013. The following key words were used: “*MTRR*” or “*MTR*”, “rs1801394” or “rs1805087”, “polymorphism” or “variant”, “Congenital Heart Disease” or “CHD”. Our search strategy combined Medical Subject Headings (MeSH) and text words, without language restriction. We also conducted a comprehensive manual check on the reference lists of retrieved articles.

### Inclusion criteria

Studies eligible for inclusion in our meta-analysis have to fulfill the following criteria: (1) the study was a case-control or a TDT design with human subjects that illustrated the relationships between *MTRR* A66G or *MTR* A2756G polymorphism and the risk of CHD; (2) the study provided necessary data to calculate odds ratios (ORs) and 95% CIs. Reviews, case reports, comments, unpublished reports and duplicate data were excluded.

### Data extraction

All data were independently extracted by two reviewers (B. Cai and T. Zhang). The following informations were extracted or calculated from the eligible studies: First author, publication year, country, ethnicity, design type of study, the number of case and controls or families, frequencies of alleles of case and control group in case-control study or the frequency of alleles transmitted from heterozygous parents to affected offspring in TDT. All disagreements were resolved by discussion.

### Statistical analysis

We utilized the ORs and 95% CIs to examine the strength of associations between *MTRR* A66G and *MTR* A2756G polymorphisms and the risk of CHD. Effect estimates were pooled using a random-effect model, and statistical heterogeneity was measured by estimating the I-squared statistic and also by implementing the Q-test; heterogeneity was considered present when *P*<0.10. To combine case-control and TDT studies, Kazeem and Farrall [28] proposed a method that calculated ORs, 95%CIs and standard errors (SEs) from independent case-control and TDT studies using a fixed-effect approach. Nicodemus [29] implemented this model, plus extended the method to the random-effects model of DerSinonian and Laird [30] in the freely-available R package Catmap (http://www.r-project.org). Sensitivity analysis was performed by omitting one study at a time and recalculating the pooled OR for the remaining studies to assess the influence of each study [31]. Publication bias was assessed by funnel plots and the Egger's test [32].

All statistical analyses were conducted using Catmap software V1.6, and *P*- values<0.05 were assumed significant.

## Results

### Characteristics of included studies

**Figure 1:**
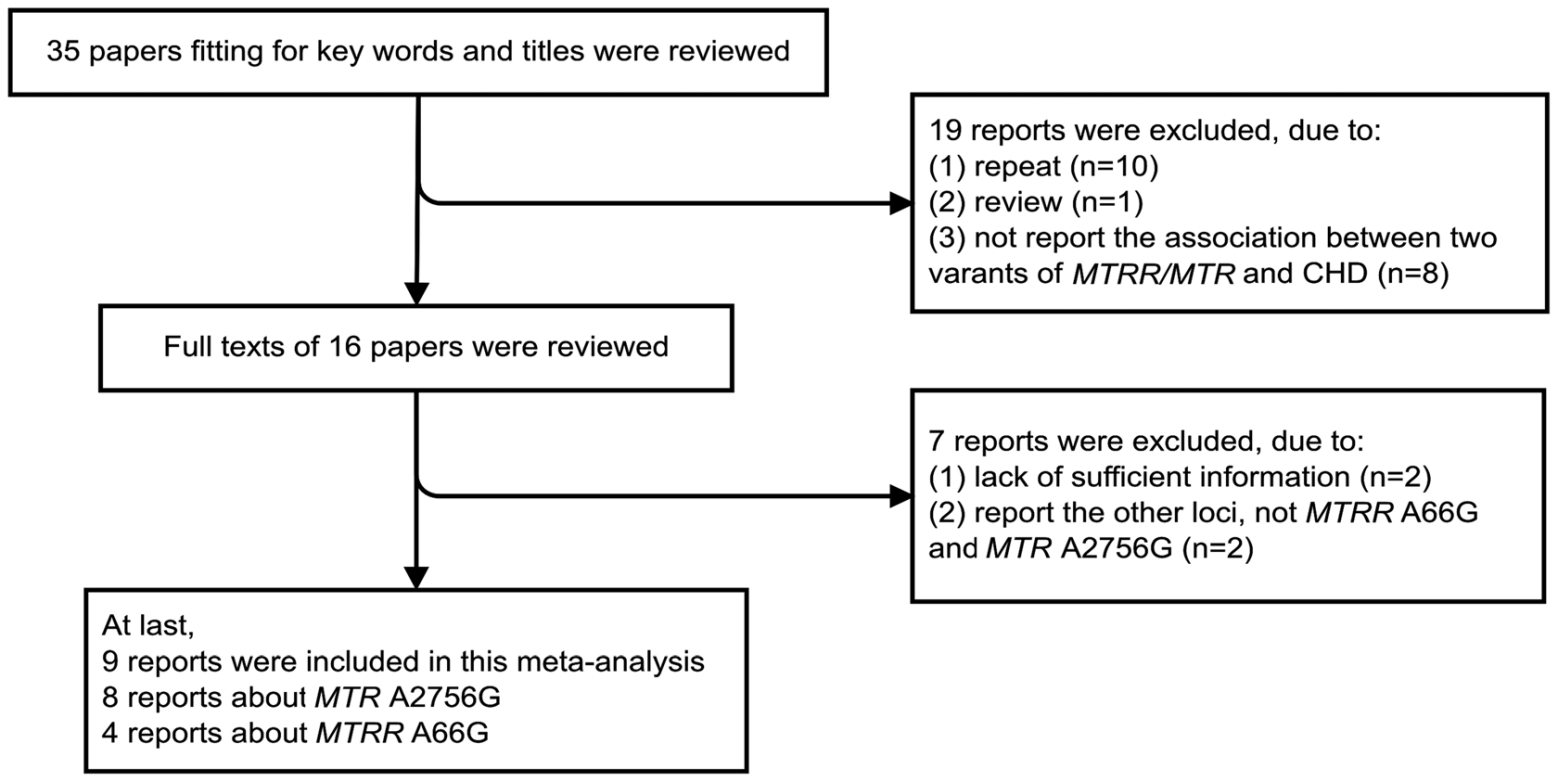
The flow chart for selection of primary studies and specific reasons for exclusion in this meta-analysis.

Figure 1 shows the literature search and selection procedures. A total of 35 papers were initially retrieved. According to the inclusion criteria, 10 repeat reports, 1 review and 8 reports without associations between *MTR* or *MTRR* and CHD risk were rejected. Among the remaining 16 papers, 5 were eliminated because of the unmatched gene loci and the studies reported by Christensen et al. [12] in 2012 and Shaw et al. [33] in 2009 were excluded due to lack of sufficient information. Finally, 9 papers were included in the current meta-analysis [19], [20], [21], [22], [23], [24], [25], [26], [27]. Furthermore, 3 reports [20], [23], [24] applied both case-control and TDT designs in the same or overlapping probands. Since the TDT eliminates concerns regarding the selection of appropriate controls and the distortion of gene frequencies due to population stratification or admixture, it is a more robust and appropriate method for validating genetic [20], [34], [35], thus the TDT were included in the final meta-analysis. Therefore, 8 reports were germane to *MTRR* A66G polymorphism including 914 cases, 964 controls and 441 families, and 4 reports were relevant to *MTR* A2756G polymorphism including 250 cases, 205 controls and 53 families. Of these 9 studies, 4 were conducted in Chinese Han, 2 in Europeans, 1 in Iranians and 2 in mixed ethnicity. All patients in these 8 studies were diagnosed based on echocardiography and cardiac catheterization. Besides, 5 and 1 studies involved all types of CHDs or specific type VSD of CHDs separately, and the rest of the studies lack of related information. Table 1 shows the characteristics of included studies.

**Table 1:** Basic Characteristics of the Included Studies.

Author	Year	Country	Ethnicity	Study design	Case/control (family)
***MTRR*** ** A66G**					
Li et al.	2004	China	Han	TDT	97
Van Beynum et al.	2006	Netherlands	Caucasian	TDT	136
Liu et al.	2007	China	Han	Case-control	132/107
Verkleih-Hagoort et al.	2008	Netherlands	European	TDT	218
Locke et al.	2010	USA	Mixed	TDT	53
Gong et al.	2010	China	Han	Case-control	60/60
Zeng et al.	2011	China	Han	Case-control	599/672
Pishva et al.	2013	Malaysia	Iranian	Case-control	123/125
***MTR*** ** A2756G**					
Liu et al.	2007	China	Han	Case-control	132/107
Galdieri et al.	2007	Brazil	Mixed	Case-control	58/38
Locke et al.	2010	USA	Mixed	TDT	87
Gong et al.	2010	China	Han	Case-control	60/60

### Overall meta-analysis between *MTRR* A66G polymorphism and CHD risk

**Figure 2:**
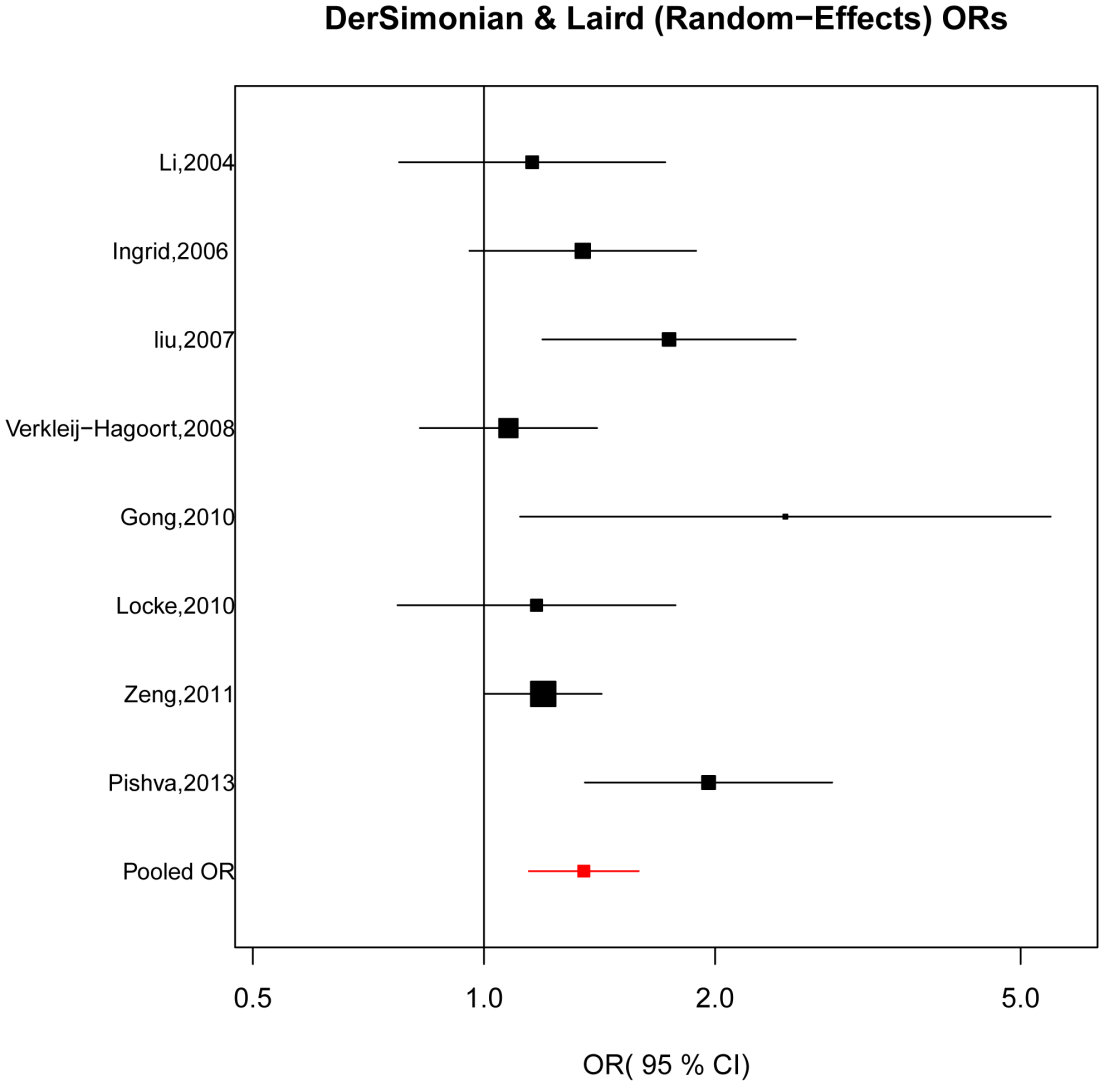
The forest plots of ln(OR) with 95%CIs for the *MTRR* A66G variant for CHD. Random-effects pooled OR = 1.35, 95% CI = 1.14–1.59, *P* = 0.00036; χ^2^ = 12.95, *P*_heterogeneity_ = 0.073.

Figure 2 shows the combined result of case-control and TDT studies for *MTRR* A66G in association with CHD. A random-effect model was employed (χ^2^ = 12.95, *P*_heterogeneity_ = 0.073, *I* ^2^(%) = 45.9) and statistical evidence indicated that the G allele of *MTRR* A66G was associated with increased risk of CHD (OR = 1.35, 95% CI = 1.14–1.59, *P*<0.001).

**Figure 3:**
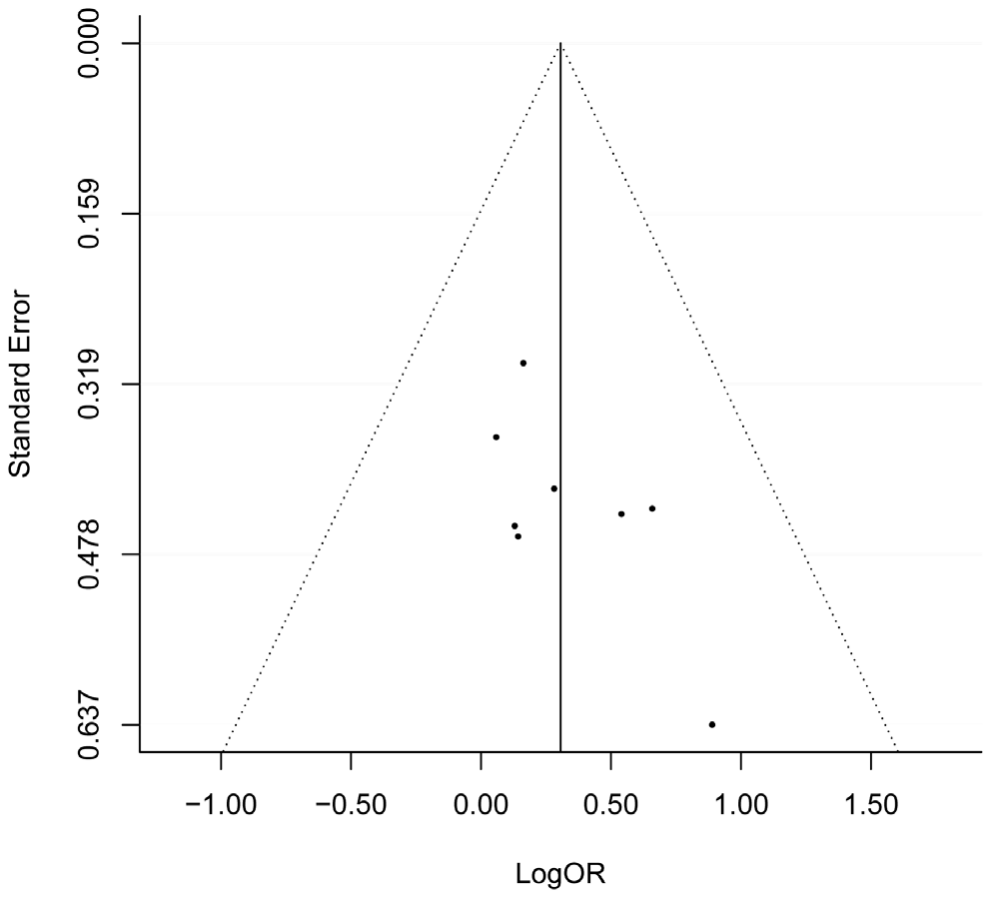
The funnel plot of natural logarithm of OR against inverse standard error in each study relevant to MTRR A66G polymorphism.

Additionally, we conducted a sensitive meta-analysis to assess the effect of each individual study on the combined OR. A series of combined ORs with 95% CIs produced repeatedly after removal of each particular study consistently exceeded 1.0, suggesting the stability of the outcome that the G allele of *MTRR* A66G may be associated with the increased risk of CHD (Table 2). What's more, Visual inspection of funnel plots suggested no evidence of publication bias (Figure 3) and no publication bias was detected by Egger's test for 66 A>G in *MTRR* (*P* = 0.291).

**Table 2:** Sensitivity Analysis of Combining TDT and Case-Control Studies for *MTRR* A66G.

Study omitted	OR (95% CI)	*P* a	?2	*P* b *_heterogeneity_*	*I* ^2^(%)
Li,2004	1.38(1.15–1.66)	0.001	12.62	0.049	52.46
Ingrid,2006	1.36(1.12–1.65)	0.002	12.89	0.045	53.46
Liu,2007	1.30(1.10–1.54)	0.002	10.37	0.110	42.12
Verkleij-Hagoort,2008	1.41(1.17–1.69)	<0.001	10.74	0.097	44.14
Gong,2010	1.31(1.12–1.54)	0.001	10.36	0.110	42.09
Locke,2010	1.38(1.14–1.66)	0.001	12.71	0.048	52.82
Zeng,2011	1.41(1.15–1.72)	0.001	11.66	0.070	48.54
Pishva,2013	1.26(1.10–1.45)	0.001	7.64	0.266	21.45

a DerSimonian and Laird Random-effects model used to determine the significance of the overall OR.

b Cochran's χ^2^-based Q statistic test used to assess the heterogeneity.

### Overall meta-analysis between *MTR* A2756G polymorphism and CHD risk

**Figure 4:**
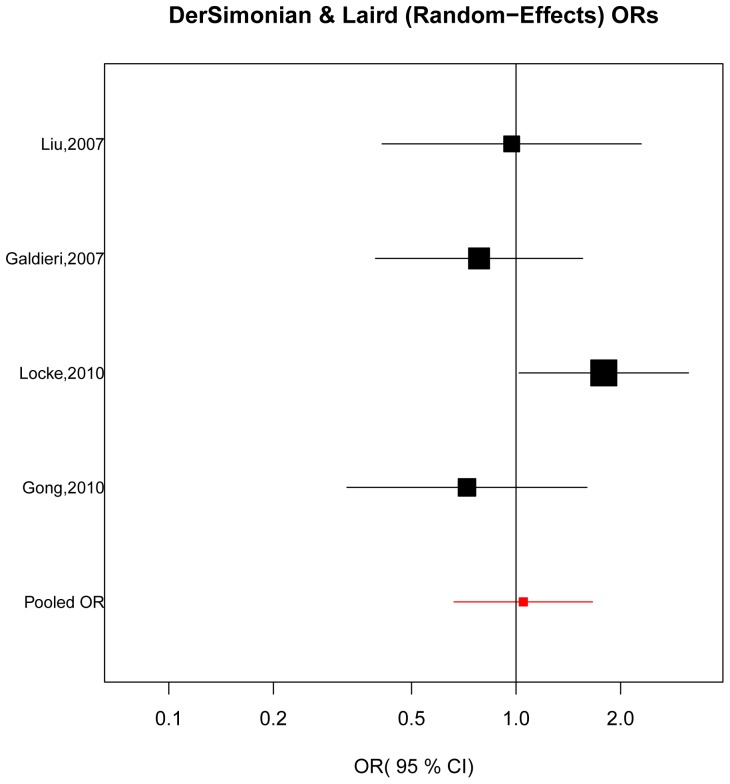
The forest plots of ln(OR) with 95% CIs for the *MTR* A2756G variant for CHD. Random -effects pooled OR = 1.05, 95% CI = 0.66–1.66, *P* = 0.837; χ^2^ = 4.99, *P*_heterogeneity_ = 0.173.

**Figure 5:**
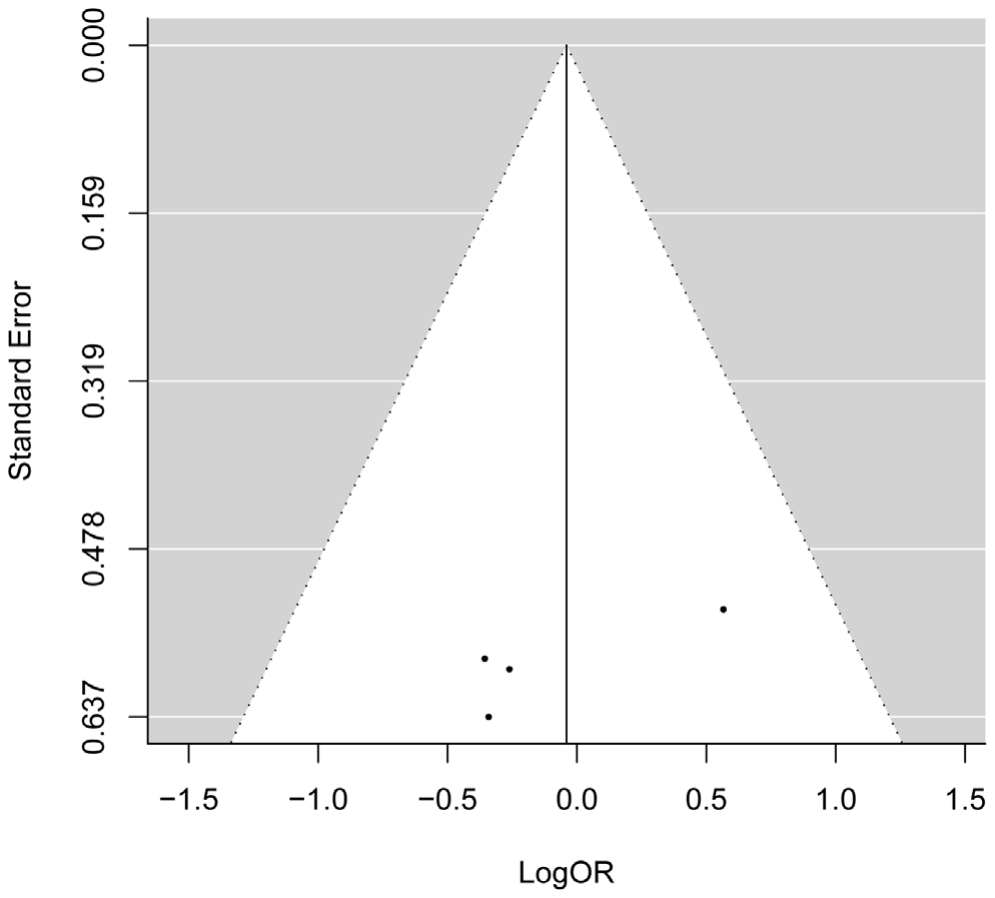
The funnel plot of natural logarithm of OR against inverse standard error in each study relevant to *MTR* A2756G polymorphism.

As shown in Figure 4, no substantial heterogeneity was found (χ^2^ = 4.99, *P*_heterogeneity_ = 0.173, *I* ^2^ (%)  = 39.8) and there was no statistical evidence of an association between *MTR* A2756G polymorphism and the CHD risk (OR = 1.05, 95% CI = 0.66–1.66, *P* = 0.837). The sensitivity analysis (Table 3) demonstrated that none of the individual study dramatically influenced the pooled OR for *MTR* A2756G polymorphism. In addition, as demonstrated by the funnel plot(Figure 5) and the Egger's test (*P* = 0.239), there was no significant publication bias.

**Table 3:** Sensitivity Analysis of Combining TDT and Case-Control Studies for *MTR* A2756G.

Study omitted	OR (95% CI)	*P* a	?2	*P* b_heterogeneity_	*I* ^2^(%)
Liu,2007	1.05(0.57–1.93)	0.878	4.89	0.087	59.11
Galdieri,2007	1.15(0.65–2.04)	0.630	3.72	0.155	46.29
Locke,2010	0.81(0.52–1.26)	0.349	0.26	0.878	0
Gong,2010	1.16(0.68–1.99)	0.591	3.66	0.160	45.42

a DerSimonian and Laird Random-effects model used to determine the significance of the overall OR.

b Cochran's χ^2^-based Q statistic test used to assess the heterogeneity.

### Stratified analysis

The stratified analysis was first performed by study design (Table 4). For the *MTRR* A66G polymorphism, evidence of heterogeneity was found in the case-control studies (χ^2^ = 9.55, *P*_heterogeneity_ = 0.023, *I* ^2^(%) = 68.8), but not in the TDT studies (χ^2^ = 3.24, *P*_heterogeneity_ = 0.794, *I* ^2^(%) = 7.4). The pooled allelic ORs were 1.64 (95% CI = 1.18–2.27, *P* = 0.003) in the case-control studies and 1.17 (95% CI = 0.99–1.38, *P* = 0.072) in TDT studies, respectively.

**Table 4:** Stratified analyses of the MTRR A66G polymorphisms in association with CHD risk.

Subgroup	OR (95%) CI)	*P* a	?2	*P* b_heterogeneity_	*I* ^2^(%)
**study design**					
case-control study	1.64(1.18–2.27)	0.003	9.55	0.023	68.8
TDT	1.17(0.99–1.38)	0.072	3.24	0.794	7.4
**ethnic**					
Chinese Han	1.29(1.11–1.49)	0.001	6.00	0.112	50.0
non-Chinese Han	1.33(1.03–1.73)	0.031	6.95	0.074	56.8

a DerSimonian and Laird Random-effects model used to determine the significance of the overall OR.

b Cochran's χ^2^-based Q statistic test used to assess the heterogeneity.

These data were further stratified by ethnicity into 2 subgroups: the Chinese and the non-Chinese subgroups (Table 4). Stratified by ethnicity, significant heterogeneity was detected in the subgroup of non-Chinese (χ^2^ = 6.95, *P*_heterogeneity_ = 0.074, *I* ^2^(%) = 56.8), but not in the other subgroup (χ^2^ = 6.00, *P*_heterogeneity_ = 0.112, *I* ^2^(%) = 50.0). The association between *MTRR* A66G and the CHD risk was found in both subgroups (Chinese subgroup: OR = 1.29, 95% CI = 1.11–1.49, *P* = 0.001; non-Chinese subgroup: OR = 1.33, 95% CI = 1.03–1.73, *P* = 0.031).

In terms of the subtype of CHD, the pooled ORs specific to each subtype could not be estimated due to limited data. Regarding *MTR* A2756G polymorphism, the stratified analysis could not be conducted due to lack of studies.

## Discussion

As far as we know, our meta-analysis first integrated the case-control and TDT studies to provide important insights into the association between *MTRR*/*MTR* variants and the risk of CHD. The results of this study may be indicative of a association between *MTRR* A66G variant and the CHD risk (OR = 1.35, 95% CI = 1.14–1.59, *P*<0.001). In addition, the stability was well supported by the sensitivity analysis. Heterogeneity was observed in the overall analysis and the stratified analysis on *MTRR* A66G indicating that the case-control study may be one of the potential sources of heterogeneity. This meta-analysis also integrated studies on *MTR* A2756G, however, no significant evidence was found.

A number of studies have demonstrated that genetic defects of these enzymes will trigger folic acid metabolic disorders, which may lead to CHD. The vast network of folic acid metabolism involves the interaction of various enzymes, requiring effective coordination of folic acid and vitamin B, which affects the folate pathway through regulation of multiple metabolism processes. *MTR* gene located at chromosome 1q43, encoding methionine synthase, which can transfer the methyl-group from 5mTHF to Hcyand produce MET(methionine) and THF (tetrahydrofolic acid) by means of remethylation [36]. It is crucial since it is the only way to reduce the level of Hcy (homocysteine) and produce MET in the early stage of embryo development. Therefore, when defected, it ultimately leads to the folic acid metabolic disorder. *MTRR* gene, located on chromosomes 5p15.31, encodes methionine synthase reducate, playing a key role inactivating methionine synthase. Thus, *MTRR* genetic defect might inhibit the development of the fetuses by changing the concentration of Hcy, 5mTHF, MET and THF [37].

Despite that no significant evidence was found in the first literature on *MTRR* A66G variant [19], several replication experiments have been conducted to explore the association between *MTRR* A66G and the risk of CHD. Zeng et al. [26] first demonstrated a relatively accurate data that the G allele of *MTRR* A66G was associated with a 1.5-fold increased risk of CHD and Pishva et al. [27] reported 1.9-fold. Similar outcomes had been previously reported by liu et al. [21] and Gong et al. [25]. However, other studies [19], [20], [23], [24] suggested that this locus has no effect on the risk of CHD. Existing studies have shown that the results were controversial.

Our meta-analysis gathered 8 reports including 914 cases, 964 controls and 441 families which indicated a suggestive association between the *MTRR* A66G variant and the increased risk of CHD (OR = 1.35, 95% CI = 1.14–1.59, *P*<0.001), supporting the results in former observations [21], [25], [26], [27]. When stratified, same results were also found between the SNP and the risk of CHD in the Chinese Han and non-Chinese Han subgroup, thus we inferred that the result of *MTRR* A66G was relatively robust in different ethnic groups, especially in Chinese Han. However, due to the small sample size of other ethnicities except Han, separate stratified analysis for each ethnicity was not feasible. Besides, no reversed results were detected in the sensitivity analysis, which showed the stability of our study.

Moreover, to explore the sources of between-study heterogeneity in *MTRR* A66G polymorphism, we conducted a comprehensive stratified analysis and sensitive analysis. There was no indication of heterogeneity among TDT studies (χ^2^ = 3.24, *P*_heterogeneity_ = 0.794, *I* ^2^(%) = 7.4) when studies were grouped according to the study design, coinciding with the result of sensitive analysis, after we omitted four case-control studies [21], [25], [27] in sensitivity analysis. When studies were stratified by ethnicity, heterogeneity reduced sharply in the Chinese Han subgroup while it was not eliminated in the non-Chinese Han subgroup. It could be argued that the evidence of heterogeneity in the case-control studies (χ^2^ = 9.55, *P*_heterogeneity_ = 0.023, *I* ^2^ (%) = 68.8) and the subgroup of non-Chinese (χ^2^ = 6.95, *P*_heterogeneity_ = 0.074, *I* ^2^ (%) = 56.8) indicated that the heterogeneity of the effects of the *MTRR* A66G across association studies may due to the differences in ethnicity and the variety of participant selection and study design in different studies. However, the small sample sizes limited us to eliminate these studies to perform further analysis. Furthermore, sensitivity analysis and the publication bias assessment indicated that the current results of *MTRR* A66G in this meta-analysis were robust.

No significant evidence of correlation between *MTR* A2756G and the risk of CHD was observed in our meta-analysis (OR = 1.05, 95% CI = 0.66–1.66, *P* = 0.837). Since the genotype is of low frequency in most populations with small effect sizes, limited sample sizes may lead to instability of the results and greater variety between studies. Additionally, since CHD is a multiple etiology disease, we cannot exclude the potential influence of gene-gene and gene-environment interaction with a relatively small sample size. The sensitivity analysis also demonstrates that the result of current meta-analysis was unstable.

In order to provide more powerful statistical evidence with robust ORs and *P* values, we combined case-control and TDT studies in this meta-analysis. Although the statistical power increased by integrating case-control and TDT studies, some limitations should also be seriously considered. Firstly, mixed population of samples, distinct subtypes of CHD patients, diverse age range, female/male ratio, control matched condition in case-control design and multiplex or simplex trios utilized in TDT design may possibly affect the current result. Secondly, CHD is a complex trait caused by both genetic and environmental factors; lacking of environmental data limited our further evaluation of gene-environment interaction. Thirdly, the difference in the allelic frequency between individuals of different ethnic populations may be a confounding factor, which affected the efficacy of the results.

Moreover, although the recent advances in genome-wide association study and subsequent replication studies [38], [39], [40], [41] have successfully genetic dissected many complex diseases, there are no genome-wide data available to be included in this study.

In conclusion, our meta-analysis suggests that *MTRR* A66G polymorphism might be associated with the risk of CHD. However, *MTR* A2756G failed to support an evidence of the association, and more studies with larger sample sizes concerning the association between the *MTR/MTRR* variants and CHD are required to confirm the current findings.

## Supporting Information

Checklist S1 PRISMA checklist.
(DOC)
